# The creation of the health consumer: challenges on health sector regulation after managed care era

**DOI:** 10.1186/1744-8603-7-2

**Published:** 2011-02-24

**Authors:** Celia Iriart, Tulio Franco, Emerson E Merhy

**Affiliations:** 1 Department of Family and Community Medicine and Robert Wood Johnson Foundation Center for Health Policy, University of New Mexico, MSC09 5060,1 University of New Mexico, Albuquerque, NM 87131-0001, USA; 2 Departamento de Planejamento em Saúde, Instituto de Saúde da Comunidade, Universidade Federal University Fluminense, Av. Marquês de Paraná, 303-Anexo, 4o. Andar, Centro Niterói, RJ, CEP: 24030-210, Brasil; 3 Instituto de Psiquiatria, Programa de Psiquiatria, Universidade Federal do Rio de Janeiro, Av. Vensceslau Brás 71, Fundos, Botafogo, 22290-040 - Rio de Janeiro, RJ - Brasil

## Abstract

**Background:**

We utilized our previous studies analyzing the reforms affecting the health sector developed in the 1990s by financial groups to frame the strategies implemented by the pharmaceutical industry to regain market positions and to understand the challenges that regulatory agencies are confronting.

**Methods:**

We followed an analytical approach for analyzing the process generated by the disputes between the financial groups and the pharmaceutical corporations and the challenges created to governmental regulation. We analyzed primary and secondary sources using situational and discourse analyses. We introduced the concepts of biomedicalization and biopedagogy, which allowed us to analyze how medicalization was radicalized.

**Results:**

In the 1990s, structural adjustment policies facilitated health reforms that allowed the entrance of multinational financial capital into publicly-financed and employer-based insurance. This model operated in contraposition to the interests of the medical industrial complex, which since the middle of the 1990s had developed silent reforms to regain authority in defining the health-ill-care model. These silent reforms radicalized the medicalization. Some reforms took place through deregulatory processes, such as allowing direct-to-consumer advertisements of prescription drugs in the United States. In other countries different strategies were facilitated by the lack of regulation of other media such as the internet. The pharmaceutical industry also has had a role in changing disease definitions, rebranding others, creating new ones, and pressuring for approval of treatments to be paid by public, employer, and private plans. In recent years in Brazil there has been a substantial increase in the number of judicial claims demanding that public administrations pay for new treatments.

**Conclusions:**

We found that the dispute for the hegemony of the health sector between financial and pharmaceutical companies has deeply transformed the sector. Patients converted into consumers are exposed to the biomedicalization of their lives helped by the biopedagogies, which using subtle mechanisms present discourses as if they are objective and created to empower consumers. The analysis of judicialization of health policies in Brazil could help to understand the complexity of the problem and to develop democratic mechanisms to improve the regulation of the health sector.

## Background

As we demonstrated in our previous study the worldwide domain of financial capital, which has been increasing since the middle of the 1970s, defined the reforms of the health sector in the decades that followed [1-5]. Insurance companies and administrators of mutual and pension funds expanded their business opportunities by not only moving into different countries but by also entering into new economic sectors, such as health. At the end of the 1980s and beginning of the 1990s the flows of financial capital into the health sector increased exponentially [1]. This process occurred first in the U.S. and after in many developed and developing countries, introducing new social actors, new rules, and new insurance models that have direct impact on the management and provision of health care services. Insurance companies had operated in the health sector before, but their operations were limited to selling life insurance policies and health insurance plans, mostly to individuals. The radical financialization of the world economy in the 1990s, supported by U.S. policies that deregulated the financial markets, opened the doors for corporate groups to intensify their operations worldwide using new non-regulated financial tools [6].

During the late 1980s and 1990s, pharmaceutical companies and health professionals were impacted by the managed care model of containing costs. The increased hegemony of financial capital in the health sector required changes in the way that business was conducted. The business model traditionally followed by health providers and the producers of drugs, devices, and equipment depends upon increasing consumption of health services and treatments. The pharmaceutical industry focused on health professionals, especially physicians, to create or increase the demand for its products. The financial groups administrating private, public, and employer-sponsored health plans have an opposite model. These companies realize more profits by cutting access to services and treatments, especially the more costly ones. For this reason, managed care organizations developed strategies to control costs using administrative procedures to limit physicians' prescriptions and referrals.

At the beginning of the 1990's, financial groups operating in the health sector introduced explicit and silent reforms following the U.S. model of managed care in several countries in Latin America, Asia, and Europe [2]. By silent reforms we mean changes in rules related to the health sector operation and/or conceptualization that most of the time avoids the legislative process and moreover, the public debate [3].

In Latin America and other developing countries, structural adjustment policies and neoliberal ideology created the context for health reforms that allowed the entrance of multinational financial companies into publicly financed programs and employer-based insurance [4,5]. Later, financial capital entered into the management of health care services: hospitals, home care, long-term care, nursing homes, etc. [7].

In the health sector the massive entrance of financial capital changed not only the *modus operandi *at the economic level but also the common sense regarding the ideas about health-ill-care. By common sense we refer to core ideas that underlie discourses on health in a specific time and society. Common sense is shared meaning that provide direction for society, and act as social cement that fills gaps and artificially softens social contradictions. Common sense shapes the subjective assessment of a shared situation by people in different places in the social structure (p. 55) [3]. Cost containment, individual responsibility, cost-effectiveness, case management of patients, coordination of care, etc. are technical concepts that penetrated the sector and deeply transformed the conceptualization of health-ill-care. Professional decisions were subordinated to administrative procedures which focused on maximizing profits masked most of the times as decisions based on scientific evidence.

From our previous study it was clear that the administrative model implemented by the financial groups controlled expenditures limiting access to health services operating in contraposition to the interests of the medical industrial complex. The next step in our research agenda was to understand if the most powerful group of the medical industrial complex, the pharmaceutical industry, has remained quiet while the corporate groups managing financial capital were increasing their shares of the health market. We observed that as a consequence of the massive adoption of managed care in the U.S. and other countries, the pharmaceutical arm of the medical industrial complex has developed health reforms, most of them without public debate, to regain market share and authority in defining the health-ill-care model [8]. These silent reforms radicalized the medicalization, now defined by some authors as biomedicalization [9]. Some reforms took place through deregulatory processes such as weakening the regulation regarding direct-to-consumer advertisements of prescription drugs in the United States. Others were developed more silently. This was the case of the hidden role, through paid experts, that the pharmaceutical industry has had in changing disease definitions, developing clinical guidelines for diagnosing new diseases or overdiagnosing others, and pressuring for approval of treatments to be paid by public systems and employer-based and private insurances. However, the most successful strategies were focused on converting patients into consumers.

In other countries, different strategies are facilitated by the lack of regulation of other media instruments, such as the internet. Disease campaign awareness and funding of medical scientific and educational activities are also used to hide the promotion of drugs. In recent years we have observed in Brazil a substantial increase in the number of judicial claims demanding that private insurance and public administrations pay for non-approved treatments, including the experimental ones. This could be a strategy to regain market power in countries with health care systems led by public administrations, not driven by the market. These strategies could have serious consequences for the regulatory process of the health sector.

The article will first present the strategies utilized by the pharmaceutical industry to regain market leadership in the United States. We consider it important to analyze the changes operating in this country because the U.S. medical model influences the conceptualization and practice of medicine in the rest of the world, especially in developing countries. Second, we will analyze some contributions from the social sciences that allow a better comprehension of the phenomenon of the creation of the health consumer as part of the biomedicalization led by the medical industrial complex. Finally, we will introduce the research that our team is conducting in Brazil analyzing if the judicial mandates obligating the public health system to financially cover new treatments are part of the strategies of the pharmaceutical industry to create the health consumer and the impact that the mandates have on the regulation of the health sector.

## Methods

The study followed an analytical approach that reinterprets studies developed by other authors regarding the reforms that the pharmaceutical industry has developed during the past 20 years. The results of our study about the managed care reforms provided the knowledge that the financial groups developed strategies to control providers of health care services in order to decrease expenditures. The study analyzed the ideological mechanisms and the practices developed by financial groups and other central actors in the health sector to change common sense about the conceptualization of health-ill-care, including the idea that patients/users should be transformed into consumers/clients [4]. In the light of these processes, new questions arose: will the medical industrial complex, and particularly the pharmaceutical corporations, counter attack these reforms to regain market power and hegemony in defining the medical model? What kind of strategies will they implement if their traditionally targeted groups (health care providers) were controlled by the strategies of the managed care groups?

To respond to these questions we used theoretical concepts and research methods in the Foucaultian meaning of "toolboxes" [10]. This resulted in the utilization of theoretical concepts and research methods from different authors, and not unique approaches with the pretension to produce completed explanations about the analyzed situation. We looked for concepts with the potential to discover new interpretations and new discourses. In this direction, we utilized some concepts that proved their analytical power in other studies that we conducted, such as common sense and silent reforms. We found new ones, especially biomedicalization and biopedagogies, which we considered have the potential to bring new meanings to the analyzed situation.

Several methodological approaches were useful for creating a dialog between different sources of data. We will mention two as the most important to our study: a) situational analysis that provides elements to map different discourses and, b) discourse analysis which we used as a deconstructive reading and interpretation of power relationships to gain a comprehensive understanding of the effects of these dynamics in creating new subjectivities [10,11]. We are not trying to provide unequivocal answers, but to reinterpret some strategies by connecting different processes that appear disconnected in the discourses elaborated by the two most powerful groups operating in the health sector -the medical industrial complex and the financial groups. We understand power as multiple forces operating in a specific situation and time. Power is related to the concept of governability in the meaning of forces operating strategically to structure new subjectivities [12].

The fundamental idea was to understand what the pharmaceutical industry was doing to counter attack the advances of the financial groups. To initiate the research process we followed Rabinow's idea of utilizing a traditional ethnographic method "to describe what is going on" (p. 236) [12]. As a first step, we defined the macro situation as the intercapitalistic disputes between the medical industrial complex and the financial groups operating in the health sector for increasing the share of the market and defining the medical model. At meso level we were interested in understanding if pharmaceutical groups were developing strategies to change regulations to impact the organization of the health sector. At micro level we wanted to learn if the pharmaceutical industry was also creating some mechanisms to recapture the supply side of the health care sector equation and/or developing mechanisms to capture the demand side (patients/users).

We developed a non-systematic data collection and analysis following the ethnographic method of observing and analyzing data/information to widely describe the situation [12]. Following Clarke, we can define our study as a multisite/multiscape research, in the meaning that we examined multiple kinds of data from a particular situation of inquiry (p. 165) [11]. We utilize data from different sources, such as articles, news, or other data from list servers, materials researched for our teaching and other research activities, as well as, data and new insights of the situation gather by participating in national and international forums related to health sector reforms. The initial materials gathered opened other pathways to obtain additional data, such as references to articles, names of companies, and individual and collective social actors involved in the situation, and description of regulations, among others. In addition, we cannot deny that our personal experiences receiving information as consumers from pharmaceuticals were also an important source of data and inquiry. As researchers we are part of the situation as subjects that produce discourses about the situation, but also subjected to discourses that others produce [13].

The extensive preliminary analysis allowed us to notice that pharmaceuticals were turning to their favor the strategy initiated by managed care organizations of transforming patients/users into clients/consumers. From the preliminary analyses we also understood that the pharmaceutical industry was taking part in a silent process of health reforms to regain market power that impact different levels: policy, economic, ideological, and social. In addition, the team was interested in understanding how the process operates at macro, meso, and micro levels. The separation into these levels had only methodological purposes; the process is intricate and operates most of the times simultaneously at all levels. We decided that the line of analysis that will allow a wide and deep comprehension of the process will be through the utilization of analytical categories that capture the reforms that pharmaceutical companies were involved in and benefit from. The broad categories that we defined were changes in regulations, changes in defining, branding and overdiagnosing diseases, and mechanisms utilized to capture the demand side. Complementing this process, part of our team started a literature review to describe the increasing involvement of the judicial branch in the regulatory process in the health sector in Brazil.

Using these categories, we developed a systematic literature and document review to find the data (primary and secondary) that describes changes in regulations and scientific norms, and in business models consistent with the creation of health consumers. With the documents identified from different sources (academic articles, books, brochures, websites of professional, patient and consumer associations, adds and news in television, magazines, and newspapers, among others), we initiated a process of mapping the information obtained in each category from different sources to describe the situation and the results of the changes in creating the health consumer and radicalizing the medicalization. We followed the procedures described by Clarke and we recommend her book to learn about the operational steps that we will not present here because an in-depth explanation of these complex methods merit a separate article [11].

The result of the process was the analytical description of selected strategies implemented by the pharmaceutical industry and their reinterpretation applying the theoretical concepts of biomedicalization and biopedagogies. This reinterpretation allows us to increase the understanding of the effects that the discourses currently modeling the health sector have in creating new subjectivities and biosocialities, some of them as part of the hegemonic discourse and others questioning it.

The study contributes to the field of health policy by highlighting how silent reforms introduced first by financial companies and after by the medical industrial complex, are challenging the regulation of the health sector. The described analytical approach allowed us to reinterpret the situation and show the need for further study of the hidden processes and social actors that could be behind the judicialization of the health sector in countries were health is considered a common good not a commodity.

## Results

### The medical industrial complex: strategies to regain market leadership

To revitalize its role in the health sector the pharmaceutical industry needed to find new strategies [14]. The strategies were selected to create a new disease/ill/care model, make drugs for healthy people, and to exploit the idea developed by managed care organizations to convert users/patients into clients/consumers [15]. In order to accomplish these goals, the industry has been successfully lobbying for regulatory changes and in developing strategies to change the common sense related to the conceptualization of health-ill-care. We will analyze some of these changes and strategies in the following sections.

#### Changes in regulations

As a first step in converting patients/users into clients/consumers, the pharmaceutical industry required some changes at the regulatory level. Several laws and modifications of existing regulations were approved in the U.S. that facilitated the transformation of the business model of pharmaceutical companies. For this article we will consider two important regulatory changes, one that facilitated the increase of the number of pharmaceutical products in the market, and another that enhanced the ability of the companies to offer the products to consumers. The first one is the act that introduced user fees to be paid by pharmaceutical companies to the Food and Drug Administration (FDA) to initiate the approval process for new drugs. The second one is related to the changes introduced on the regulation of the direct-to-consumer advertisements.

##### Introducing user fees for drug approvals

In 1992, the U.S. Congress approved the Prescription Drug User Fee Act (PDUFA) with relatively little public debate [14]. The Act allowed pharmaceutical companies to pay fees to speed the approval process and the FDA the ability to use these fees to finance the specific areas. After the Act was launched, new approvals were being issued in unprecedented numbers and review times were at historic lows. Important new drugs for cancer, AIDS, heart diseases, and stroke were approved using this process, benefiting many patients. But also numerous new drugs for chronic diseases, mental health and lifestyle conditions, some of them not very useful or really new and sometimes dangerous, were approved, opening a new dimension for the pharmaceutical business. According to Angell, in 2002 the FDA approved 78 drugs. From those only 17 contained new active ingredients and just seven were classified by the agency as improvements over existing drugs (p. 16-17) [16]. The speedy process established in the U.S. determined that many pharmaceutical companies chose this country for the introduction of new drugs into the market more than any other country in the world.

The approval of this Act has also had consequences in other countries. The most important consequence was that in 1990s the World Bank (WB) and the Inter-American Development Bank (IADB) promoted, in several Latin American countries, the creation of regulatory agencies following the FDA model, especially the user fee provision. The reasoning of the WB and IADB was that the user fees will counter the fiscal deficit by generating revenues to finance the new governmental agencies. Additionally, according to these international lending agencies, the efficiency of the bureaucratic process of drug approval will be improved. Argentina (1993) and Brazil (1999) created the agencies to regulate drugs, foods, and medical technologies following the FDA model of charging pharmaceutical companies fees to initiate an approval process [17,18].

##### Changing the rules for advertising drugs

The new regulation speeding the approval process increased the amount of pharmaceutical products in the market, but additional strategies were required to increase the demand of the products. The capture of the supply side (providers) of the health care equation developed by managed care organizations, mobilized pharmaceutical companies to develop strategies to capture the demand side. The industry envisioned the idea to convert patients into consumers with the right to be informed about pharmaceutical products as they have the right to receive information about other goods and services [14].

For years medical associations and also pharmaceutical executives opposed direct-to-consumer advertisements (DTCA) of prescription drugs, arguing that it will interfere in the physician-patient relationship. But during the 1980s the increasing power of consumer organizations created the possibility of advertisements as a way to empower patients [14]. This idea was captured by the managed care industry to promote the concept that an informed patient could make rational decisions about their health needs and not be manipulated by prescribers. Using this concept, innovators within the pharmaceutical industry convinced those who initially questioned that the DTCA of prescription drugs was a way to empower patients. Also, they promoted the idea that the DTCA will create more awareness about diseases and conditions not well known, but which were silently affecting huge number of people.

DTCA already was approved and regulated by the FDA, but the requirements were that the advertisements must present the entire list of side effects and cautions, limiting the power of the promotion by scaring the public. In 1997, the FDA rolled back this requirement and it opened the way for a new era in DTCA [16]. Companies increased exponentially the amount of DTCA. In 2001, the big companies spent $1.8 billion in DTCA, but in 2003 they spent $3 billion [14]. The regulatory capacity of the agency is almost null to control this increased amount of advertisements, especially if we consider that in 2001 drug companies also substantially increased the ads in newspapers and magazines, exceeding largely the number of ads in medical journals for that year. Drug companies are required by law to have their DTCA reviewed by the FDA when they launch a new campaign. However in 2001, the agency had only 30 reviewers to control 34,000 DTCA. In addition, the agency cannot verify whether the companies submit all the ads [19].

Most of the DTCA are for chronic and lifestyle conditions, as well as behavioral/mental health problems. The ads will train consumers about signs and symptoms, as well as brand name medications, and direct them to consult with their physicians. Perhaps, it would be more appropriate to say, to convince their physicians about the diagnosis and treatment. These advertisements are now the biggest investment of pharmaceutical companies in marketing and include ads in television, radio, magazines, internet, and mail advertisements, among others. But more than providing useful information to patients for specific health problems, the ads market the diseases/conditions (old, new, and rebranded) for which the pharmaceuticals have drugs to sell. The idea, promoted by the new pharmaceutical paradigm, is not just to sell pills to cure diseases, but to create a state of fear of becoming sick, aging, and dying [15,20].

In other countries, pharmaceutical companies are pressuring regulators to allow DTCA for prescription drugs and to modify the classification of some drugs to be sold over-the-counter. While drug companies are waiting for regulation changes, they are using other strategies to reach consumers, such as media disease awareness campaigns, promotion of drugs presented as news in television and other media, and developing health teaching materials for schools, among others. The central idea is to increase the capacity of consumers to self-diagnose and self-prescribe [21,22].

#### Changing health definitions, branding diseases and overdiagnosing others

Since the middle 1990s, pharmaceutical companies have had more influence within the expert committees reviewing disease/condition definitions and describing new diseases and health risks to be covered by private, employer-based, and public insurance plans [16]. The majority of these changes are defined by medical specialists in committees organized by official agencies, professional organizations, and international institutions, like the World Health Organization (WHO). Most of the time, the decisions of these bodies of experts, presented as based on scientific research, are adopted worldwide.

In middle 1990s, committees of experts redefined several diseases/conditions and health risks. These committees decreased the parameters for high blood pressure and defined prehypertension; decreased the level of fasting glucose to define diabetes mellitus and of serum cholesterol for hypercholesterolemia; also experts decreased the Body Mass Index values for being overweight and obese [16,23]. In the U.S., these changes resulted in the following increases in potential cases: 14% for diabetes; 35% for hypertension; 86% for hypercholesterolemia, and 42% for overweight. This opened the possibility for 140,630,000 additional individuals to be diagnosed with some of these diseases/conditions and treated with drugs and other medical interventions [23].

This is a dangerous approach that implies moving the bell curve of a population to the left in order to capture more people with low risk to prevent them from developing the disease/condition. While this approach may be useful for preventing exposure to hazards, such as tobacco or contaminants, to use this approach for identifying people at risk for conditions that require medications creates a potentially dangerous situation exposing healthy people to adverse effects of drugs [24]. The idea of health promotion has been deeply medicalized because healthy behaviors are now related to the capacity of individuals to control their risk of becoming sick. The social, economic, and political factors that create health risks and diseases are denied under the renewed logic of a positivist science. This logic reinstalled biological factors as the central causes for diseases, and medical procedures and individual behavior changes as the only way to cure or alleviate them.

The described changes in definitions of diseases/conditions were not the only targets of pharmaceutical companies. These companies moved from promoting drugs to treating diseases, to promoting diseases/conditions to fit their drugs. During the 1980s and 1990s companies in the U.S. started promoting lifestyle drugs, including those for cosmetic and sexual enhancement. The crossover to curative medicine occurred with psychotropic drugs that have a wide range of active properties, allowing pharmaceutical companies to expand the spectrum of diseases/conditions for which the drugs could be promoted. As Applbaum explains,

"one class of antidepressants, the specific serotonin reuptake inhibitors, is marketed for eight distinct psychiatric conditions ranging from social anxiety disorder to obsessive-compulsive disorder to premenstrual dysphoric disorder. And 'lifestyle marketing' has now extended to the promotion of many blockbuster 'maintenance drugs' intended for daily, lifelong consumption, such as drugs for allergies, insomnia, and acid reflux disease" (p. e189) [25].

Previously, acid reflux was known simply as heartburn and treated with a glass of milk or an over-the-counter antiacid. In the 1990s in the U.S., Glaxo began promoting one of its drugs to treat heartburn under a new name, GERD (gastroesophageal reflux disease), and described it as having serious health consequences if not treated (p. 86) [16].

Social phobia disorder, which is a very rare condition, was renamed social anxiety disorder by SmithKline just after September 11, 2001. The company launched an ambitious campaign promoting its antidepressant Paxil for this use. The commercial showed images of the World Trade Center towers collapsing (p. 88) [16]. Corporations are branding social, cultural, and political situations as diseases, defining them as conditions that could be medicated. The list is long and includes attention deficit disorder, not only in children but now also in adults, depression, erectile dysfunction, female sexual dysfunction, bipolar disorder, restless legs, social anxiety, and panic attack, among many others.

In addition to their promotional efforts to create public awareness of new and old diseases/conditions, pharmaceutical companies create short questionnaires to test the risk for diseases/conditions. These questionnaires are extensively utilized in primary care settings by physicians, physician assistants, and nurse practitioners, especially for diagnosing mental health problems for which they are not very well trained to diagnose. Using these tools, non-specialized professionals feel confident about their diagnoses and their capacity to medicate adults and children with psychiatric drugs. The strategies implemented by pharmaceutical companies successfully expanded the market through increasing the amount of professionals diagnosing and prescribing. This strategy, together with the increased investments in directed-to-consumer marketing generated the results for which they were developed. The top 25 drugs directly marketed to consumers in the U.S. rose by 34% from 1998 to 1999, while other prescriptions rose only 5.1% [9].

#### Capturing the demand side

Another strategy that pharmaceutical companies utilized, initially in the U.S., to regain market positions was to support patients and consumer associations. The pharmaceutical groups learned from the women's health movements and AIDS patient groups how valuable advocates could be for demanding increases for research funds, the approval of new drugs, and the coverage of new treatments by public programs, and employer-based and private health plans [9].

Pharmaceutical companies targeted patient and consumer organizations offering funds to help them to create awareness of diseases/conditions in the general public and among policymakers [14]. Education was the new strategy to a) pressure health plans to pay for costly drugs, especially new drugs or off-label indications of approved ones, most of the time with not much more benefit than the older ones but more expensive; b) discredit some treatments, such as psychotherapies, as costly and not as efficacious as new drugs for treating mental health disorders; and c) convince health plans about the improvement outcomes of some drugs beyond the specific indications, e.g. a drug like Fosomax was not only presented as a bone density builder but as a breakages preventer [15].

The strategy proved to be successful for the pharmaceuticals. Patient and consumer organizations were powerful allies for pressuring the Congress for regulatory changes needed by the pharmaceutical industry [14]. Patient associations have demonstrated to be good allies in pressuring for approvals of new drugs and for classifying new diseases and conditions to become recognized, and consequently, to be reimbursed by private and employer-based health plans and public programs. Patient testimonies have been very useful in convincing the FDA scientific reviewers to approve new drugs, despite the fact that in some cases serious concerns about drug safety existed. Also these groups have been very instrumental in pressuring reticent physicians to prescribe drugs for off-label uses. In the U.S., pharmaceutical companies cannot promote off-label use of their products but can inform physicians about them. Patients, well trained by the industry, are the best way to create the demand for these off-label treatments [16]. Patient advocacy organizations that offer lists of physicians to patients and also recommend treatments become powerful allies of the pharmaceutical industry [14].

Awareness campaigns and health fairs in school and community settings also are tools that the companies have utilized since the middle of 1990s. In 1994, Eli Lily sponsored a National Depression Awareness Day in a Maryland high school, reaching 1,300 students [14]. Other companies followed this initiative on college campuses. The awareness campaigns commonly go a step further by promoting the use of the drugs that the sponsor makes. More importantly, they market the condition, teaching about very general signs and symptoms that mobilize people to request medical help [16]. For their awareness campaigns, as well as for television shows, ads, and other types of promotions, the companies also contract celebrities to talk to the public about their struggles against some diseases and how medications have helped them to live successful lives [16,26]. Health fairs in community settings also offer a good opportunity to expand the number of potential consumers of pharmaceutical products. Companies offer free tests for cholesterol, diabetes, high blood pressure, etc. and information about these diseases/conditions. Another interesting way that pharmaceutical companies use to create awareness of diseases and treatments is to post communication tools on their websites to facilitate the "dialogue between physicians and patients." Some tools are for patients and others for physicians [27].

Pharmaceutical companies create new tools to facilitate the identification of potential consumers of their drugs. Short questionnaires to test the risk for diseases/conditions are available in magazines, as well as at websites of pharmaceutical companies, patient associations, and associations for specific diseases (e.g. American Diabetes Association) [26,28]. Moreover, pharmaceutical companies also send questionnaires by mail to potential consumers with information about specific drugs. Additionally, these questionnaires are available in primary care settings and schools. The questionnaires contain a few general questions about common signs and symptoms. Depending on the amount of positive answers, the respondent is recommended to consult with a doctor. Despite the fact that the test may conclude that a person is not at risk, the recommendation is that he/she should learn more about the disease/condition because the questionnaire may not have evaluated all risks [28].

Some pharmaceutical companies fund cable television channels dedicated solely to provide health information. Special programs and the technology to watch them are offered free to hospitals and have been installed in patient areas. The programs combine specific information with ads and logos of the company that sponsor them [16]. Moreover, health news segments and health information on television are also new ways to inform consumers about research findings or to create awareness of diseases/conditions and the availability of treatments. Pharmaceutical companies produce this information that is then offered free of charge to the media [29].

Other companies created subsidiaries to develop disease management services for managed care organizations and big employers that manage their own health insurance plans, such as Ford and General Motors [14]. The service consists in providing the health plan members with state-of-the-art information about disease/conditions and how to maintain medication compliance. The websites also offer tools to engage patients on healthy behaviors, such as exercise and nutrition. The sites provide people with health toolboxes that allow them to register doctor appointments, medication times, and medication renewal dates. The websites would then prompt patients to comply with their treatments and check other biological and behavioral indicators (blood pressure, weight, nutrition, physical activities, among others), and to enter the data to monitor improvements.

These databases provide manager companies a large quantity of information about demographic profiles of how certain patients are prescribed, use medications, and other health data. Companies are able to perform sophisticated data mining to understand patient and physician behavior in regards to a specific health problem or condition and consequently develop more precise promotion of their drugs. For Merck, a pioneering company in developing this type of information management capabilities, sales increased 23% in 2000. The subsidiary created by Merck, Medco, managed the pharmaceutical benefits of 51 million Americans in this year [14].

In the following years, free and paid websites of this kind flourished, especially for chronic diseases, lifestyle conditions, and mental health disorders. Subscriptions to these websites opened the doors for multiple companies and organizations (not only pharmaceuticals) to send information about diseases/conditions, and to promote drugs, diet products, medical procedures, etc. The worldwide capability of these tools allows companies to operate beyond the geographical boundaries and avoid regulations in place in specific countries.

## Discussion

### Biomedicalizing life to regain the hegemony of the health sector

In previous sections we analyzed the strategies utilized by the pharmaceutical industry to regain market positions and leadership in defining the health-ill-care process in the U.S. From the U.S., multinational companies export, through their subsidiaries in other countries, to the rest of the world their new business models and strategies to regain market power. Models and strategies that the companies modify accordingly with the health sector organization and the regulatory environment in each country. Next we will consider some concepts from social sciences theories to better understanding the deep implications of these strategies for the lives of people.

Medicalization is a concept developed in the 1970s to define aspects of life as medical problems previously outside of the jurisdiction of medicine [30-32]. Profound transformations in medicine, facilitated by advances in technosciences, started in the 1980s. To understand these transformations Clarke et al. developed the concept of biomedicalization, based on Foucault's theory of biopower and later developments by Rainbow [9]. While medicalization focused on illnesses, rehabilitation, and care; biomedicalization focuses on health as an internalized moral mandate of self-control, surveillance, and transformation. Advances in biomedical technosciences, such as molecular biology, genomization, medical diagnostic and treatment technologies, as well as computer and communicational developments created the possibilities for radicalizing the medicalization. Biomedicalization implies a "shift from enhanced control over external nature (i.e. the world around us) to the harnessing and transformation of the internal nature (i.e. biological processes of human and non-human life forms), often transforming life itself" (p. 164) [9]. Biotechnologies, including drugs and other devices available to patients/consumers, create new biomedicalized subjectivities, identities, and biosocialities. New forms of social relationships are constructed around and through such identities (p. 165) [9]. Examples of these kind of new forms of social relationships are the social networks using websites, blogs, and other internet forums dedicated to health issues. It is important to remark that these new identities do not always imply the acceptance of the biomedicalized discourses and practices: some of these forum/groups are questioning the moral mandate and other forms of biomedicalization [33]. The process is not unidirectional and different discourses are created by a multiplicity of individuals and organizations. New organizational developments and regulatory measures could transform the current situation.

The transformation of medicalization into biomedicalization required the confluence of several processes in which the financialization of the health sector had an important role. This process increased exponentially the corporatization of the sector and the commodification of their products at different levels: provision, research, and education. These changes mobilized others stakeholders such as the pharmaceutical industry to develop new strategies. Some of these strategies, as we analyzed previously, included enhancing the direct relationship with potential clients of health products (diagnostic procedures and treatments), as well as health promotion and prevention programs and services.

The technoscientific medicine and its subfields, such as public health, have developed illusory discourses in which death not only could be postponed, but also prevented [20]. The dream of being eternally young with plenty of energy penetrated all social, gender and age groups. In order to obtain this goal, the message is that people should exercise strict control and surveillance over the risks that could threaten their lives. Moreover, the messages about health-ill-care are presented as social/moral mandates, meaning that if individuals are not proactively controlling their health, the results of their behaviors are at a great cost to society. This goes beyond using specific medical interventions to recover health from illnesses or diseases; it supposes the biomedicalization of health promotion and prevention, requiring the internalization of the social mandate of being healthy, and of surveillance practices at the individual level.

To be healthy in the context of biomedicalization implies that it is each individual's responsibility to test for diseases/conditions, and to utilize drugs, devices, and other technomedical products and services to control the risk of developing diseases or aggravating a condition. The services to accomplish the social/moral mandate include internet tools and other communicational mechanisms that introduce information through the intimacy of personal computer, home entertainment devices, as well as school and other small environments. Individuals are taught about diseases/conditions, how to test for them, and how to access services and products to preserve their health.

In the context of biomedicalization health professionals, in their traditional role as leaders of the process of cure or alleviation of disease, are more and more dispensable for the new business model developed by the pharmaceutical industry. As we described previously the advances in computerization and data banking facilitate the capture, storage, and analysis of enormous amounts of data from individuals. All of this information is utilized to generate messages to reach millions of people interested in health issues. The communication strategies can be developed considering differences in social classes, ages, genders, nationalities, cultures/ethnicities, diseases/conditions/risks, and so forth. There are numerous products and services that the pharmaceutical industry promotes under the concept of "educating people" to prevent the risks of becoming ill that do not require health provider mediation for their consumption. Also, we have described previously that pharmaceutical companies developed tools to teach patients how to direct their physicians and other health professionals to prescribe the desired product. Young people are especially vulnerable to these kinds of marketing messages that promote self-diagnosed and self-prescribed behaviors [21,22].

The concept of biopedagogy is also useful to complement the understanding of the analyzed phenomena. This concept, drawing from Foucault's biopower theory, is described by Wright as the normalizing and regulating practices traditionally reserved to schools, but currently appropriated by other learning and communicational spaces, and disseminated more widely through the web and other forms of media [34]. Biopedagogies place individuals under constant surveillance and towards increased self-monitoring by elevating their knowledge around diseases/conditions, as well as learning how to be healthy. Using Berenstein, Wright states that we are now living in "totally pedagogized societies" where methods to evaluate, monitor and survey the body are encouraged across a range of cultural practices (p.8) [35].

Individuals are offered a number of ways to understand and change their behaviors, as well as encouraged to take action to educate other members of their families and communities to have healthy lives. Most of the pedagogical tools are created to govern bodies and to provide the social meanings by which individuals come to know themselves and others but not the social-political environment. Moreover the scientific truths are recontextualized in different social and cultural sites to inform and persuade people on how they should understand their bodies and how to live their lives. In this light, health information is developed to facilitate the incorporation of the "outside" world (the social and economic wellbeing of others) into the "inside" (psyche and body) of the individual (p 49) [36].

In principle more access to medical and health knowledge and information could be considered democratic and it needs to be welcomed. However, in practice we should critically analyze how the data is created, by whom, and what are the interests behind this information market. In addition, the commodification of this process in capitalist societies implies that the access to information is stratified, non-democratic, and differentially affecting social groups and countries. People from the lower classes receive messages reinforcing the social/moral mandate to control their health in order that they do not become a burden to society. For these populations the biopedagogies are implemented at schools, health fairs, community events, and media (especially television). Well intentioned professionals working for public health agencies, schools, and non-governmental organizations also reproduce these messages and biopedagogies without understanding how they operate and the consequences for the biomedicalization of life. People will use their limited income in testing for glucose, cholesterol, high blood pressure, and other conditions and accessing treatments (conventional or alternative) to control these risks. However, as we can observe in health statistics, disadvantaged groups will fail to reach the healthy outcomes of the upper classes, instead they will be left with the guilt of not eating healthy, exercising, and having non-stressful lives. The messages hide that most of the health problems of these groups are not caused by their bad genes triggered by inadequate lifestyle habits, but by the unequal distribution of wealth.

### Judicialization of health policies in Brazil: a new path on the creation of the health consumer?

In this section, we will introduce the research that we are initiating in Brazil to investigate if the processes of capturing the demand side are operating through the judicialization of health policies and how they are affecting the capacity of the governmental administrations to regulate the health sector.

The importance of understanding if the judicialization of the health policies in Brazil is another strategy that the pharmaceutical industry is using to regain market power resides in two important facts: 1) in this country health is considered a common good not a private one; and 2) the governmental agencies led the regulatory process in a public-private environment of health care service provision. Brazil approved in 1988 a constitutional amendment declaring that health is a right that the state must guarantee and that each Brazilian has the right to universal and integral access to health care. The constitutional nature of the right to health opens the option for citizens to utilize the judicial branch to demand the fulfillment of their rights when they believe that the constitutional mandate is not followed.

Most of the health care services under the Brazilian Unified Health System (UHS) are offered through an extensive network of public primary care clinics and hospitals, as well as public health programs managed by municipalities. Brazilian citizens also have the right to receive health care services, paid with public funds, through private providers, if the public system does not offer the needed services. In part, because this private-public arrangement to guarantee the constitutional right to universal access to health, the public system is confronting increasing costs not matched by sufficient budget allocations. This obligates governmental administrators to deny access to some health care services and put limitations on the kind of services that can be provided. In particular, these limitations affect specialized and costly treatments, most of which are offered by the private sector. By appealing to the constitutional right, an increased number of individuals are interposing judicial claims demanding that the public system covers health care services that their physicians recommend to them.

Data from the Superior Tribunal of Justice in Brazil indicate that in 2001 only two claims related to health issues were interposed. However, by 2004 the number of cases increased to 672 [37]. The Health Secretary of Rio de Janeiro started recording the numbers of judicial claims in 1991. Since then and until 1999, the number of cases slowly increased, especially in relation to some diseases. Starting in 2000, the number of cases requiring the Justice to obligate the state to pay for procedures to treat different health problems denied by public medical institutions increased exponentially. At the end of 2002, the Health Secretary counted 2,733 judicial actions against the State of Rio de Janeiro. In March 2006 the number of claims rose to 7,758 [38].

In the majority of cases, the judges ruled in favor of the citizens, obligating the public health system to pay for the demanded services [39]. To include more medications, diagnostic procedures, and treatments to be paid by the public system is in accordance with the constitutional rule to guarantee the health right, but it creates a conflict between the health and the judicial systems. Officials from health agencies consider that the judicial branch is assuming authority for decisions that need to be made by health specialists. They complain especially when the solicited medications, treatments, or diagnostic procedures are new, experimental, or off-label indications, implying that the efficacy and safety are not well established.

The judicial branch is playing a more central role in defining the health policies by focusing only on the constitutional right. But this branch of the government may not be considering the powerful actors that could be using the justice system to mandate the inclusion of new treatments to be paid by the UHS. Moreover, increasing the number of services provided with limited funding allocation may threaten the constitutional right itself. Fewer people may be accessing basic needed services, favoring only small numbers of people that receive costly specialized treatments. In addition, if the treatments the judicial system mandates the UHS to pay are predominantly those offered by private providers, the public system is subsidizing them, when public hospitals and clinics are confronting increasing budgetary restrictions. In fact, the judicial claims appear to benefit more the middle and upper classes. A study analyzing 2,927 judicial claims demanding the state of São Paulo to pay for medications not covered by the UHS, shows that 73% of claimants reported that they live in high and middle income neighborhoods, while only 27% live in low income areas. Moreover, 74% of the cases were represented by private lawyers while only 26% were represented by public defenders [39].

These are the nature of the problems that our team is starting to investigate in Brazil. Our empirical work will focus on understanding the implications for regulatory agencies of the judicialization of the health sector, as well as the role that the medical industrial complex plays in this process. We are also studying if the Brazilian pharmaceutical industry is adapting the strategies observed in the U.S. which turns users/patients into consumers by marketing new treatments, e.g., those approved but not covered by the public system, off-label indications, or not approved at all by the regulatory agencies in Brazil. In situations like Brazil where health is a right guaranteed by the constitution, unneeded consumerism of health services promoted by the medical industrial complex may be seriously threatening the stability and continuity of the public health care system, as well as increasing health inequities. We are only at the beginning of this research agenda that will provide important information to improve the regulation of the health sector and demonstrate the need for a close dialogue among branches of the government.

## Conclusions

In this article we have analyzed how the leading force in the biomedicalization process --the pharmaceutical industry-- has developed strategies to increase their share of the health market. The dispute for the hegemony of the health sector between financial companies and the medical industrial complex has deeply transformed the sector. The big contenders (financial and industrial capital) obtained a part in the distribution of the market and economic surpluses. Public systems and employer-based insurances, as well as health workers (professionals and non-professionals), and moreover the people, are those affected by the transformations. The regulation of the sector had become largely complex. We are in the presence of an intense process of control and regulation of bodies "from inside out" as a type of biomedical governance (p.181) [9]. Patients converted into consumers are exposed to the biomedicalization of their lives helped by the biopedagogies that operate through subtle mechanisms which present information as objective and created to empower consumers. Regulatory agencies in developed and developing countries lag behind in their capacity to regulate data gathering and communication tools that the multinational corporations are creating to directly reach the population.

However, it is important to remark that we consider biomedicalization as a contradictory process that arose as part of intercapitalistic disputes and has multiple allies and opponents. The powerful forces that create biomedicalized discourses operate in a situation where other discourses try to deterritorialize, desestratify, and create "lines of flight" opening the possibility for new situations to emerge [40]. Our intention in analyzing the process is to contribute to the multiplication of more democratic voices.

We recognize that the medical model dominated by the technosciences is not the only one operating around the world. Other models, more centered on the needs of the patients than on the consumption of technical procedures, are also operating and disputing the conceptualization of the health-ill-care model. It is especially true for countries such as Brazil, with a successful recent history of creating a public health care system to guarantee the right to health of its population. However, we also observe that the interests linked to the biomedical model based on technosciencies are pressuring the regulatory agencies to change the model so that these interests can regain power. Efforts to improve the regulation, as well as new mechanisms, should be developed.

Numerous researchers and activists are creating and spreading useful information for people making informed decisions about their health and to discriminate between their individual problems and those generated by the organization of societies. The analysis of judicialization of health policies, as we are observing in Brazil, could help to understand the extension and complexity of the problem and help to create a dialogue between professionals working in health and judicial systems. Public institutions should generate and distribute scientific, non-biased health information and alert the population about the biased websites and other sources of data gathering and information spreading that are paid by private groups to pursue their commercial interests. The regulation of this type of communication and the means to transmit the messages should be strengthened, but it will require a profound and broad debate within each country. Regulating information is a delicate matter. Regulation could be a good instrument to protect the people from spurious commercial interests, but also it could be used to decrease the voices that oppose the biomedicalization. Councils integrated with members from community groups, professional associations, health advocate organizations, among others, could be a tool to advise and control governmental regulatory agencies. Professional and consumer associations, publicly funded and supervised, could increase the amount of non-biased information. All efforts need to have the objective to increase the visibility of discourses defending the individual and collective right to live healthy lives non-governed by self-surveillance and consumption of bioproducts, but more connected with the need to collectively improve working and living conditions.

## Competing interests

The authors declare that they have no competing interests.

## Authors' contributions

CI contributed to the overall study's design; gathered, analyzed, and interpreted the data; and drafted the article. TF and EEM contributed to the study's design, gathered, analyzed, and interpreted the data regarding the judicialization of health sector in Brazil, and to interpret the entire data. All authors read and approved the final manuscript.
